# Biphasic changes in spontaneous cardiovagal baroreflex sensitivity during passive hyperthermia

**DOI:** 10.1038/s41598-019-39172-8

**Published:** 2019-02-22

**Authors:** Marian Turcani, Elham Ghadhanfar

**Affiliations:** Department of Physiology, Faculty of Medicine, Kuwait University, P. O. Box 24923, Safat, 13110 Kuwait

## Abstract

Successful adaptation to passive hyperthermia requires continual adjustment of circulation, which is mediated mainly by the autonomic nervous system. The goal of this study was to explore the alterations in spontaneous cardiovagal baroreflex sensitivity (BRS) during exposure to a hot environment. To continuously follow changes in core body temperature (Tc), haemodynamics, and BRS, male Wistar-Kyoto rats were implanted with telemetric transmitters. BRS at an ambient temperature of 23 °C was not steady but oscillated with a maximum power in the range of 0.02–0.2 Hz. Exposure to hot air immediately shifted the distribution of BRS to higher values, although Tc remained unchanged (37.2 (0.3) °C), and the average BRS changed from 1.3 (0.3) to 3 (1.4) ms.mmHg^−1^, p < 0.0001. The degree of initial cardiovagal baroreflex sensitization explained 57% of the variability in the time to the onset of arterial pressure decline (p = 0.0114). With an increasing Tc (>38.8 (0.6) °C), BRS non-linearly declined, but haemodynamic parameters remained stable even above a Tc of 42 °C when the cardiovagal baroreflex was virtually non-operative. Abrupt full desensitization of the cardiovagal baroreflex with a muscarinic blocker did not induce arterial pressure decline. Our data indicate that a progressive decrease in BRS during passive hyperthermia does not induce haemodynamic instability. The positive association between initial cardiovagal baroreflex sensitization and the time to the onset of arterial pressure decline may reflect the potential protective role of parasympathetic activation during exposure to a hot environment.

## Introduction

Dysfunctional baroreflex control of circulation may be involved in hypotensive episodes in humans during exposure to hot environments^1^. Rats with a non-functional baroreceptor reflex due to sinoaortic denervation exhibit a 4-fold decrease in thermal tolerance^2^. Consistent with this finding, improved baroreflex sensitivity was shown to protect against heatstroke^3^. However, despite extensive research, the effects of elevated core body temperature (Tc) on baroreflex sensitivity have not been definitively elucidated, particularly those of high Tc levels. While baroreflex control of vascular and splanchnic sympathetic activity seems to be either unchanged or elevated during heat stress^1,4,5^, reports on cardiovagal baroreflex sensitivity (BRS) are more variable.

Cardiovagal BRS has repeatedly been reported to be reduced in hyperthermic conditions^1,3,4^. However, the consequences of this thermal desensitization of baroreflex control of the heart rate are interpreted differently. Massett and colleagues^4^ showed that cardiovagal BRS assessed with the Oxford method in rats at a Tc of 41.5 °C was reduced, and baroreflex control of splanchnic sympathetic activity was not attenuated. According to these authors, the loss of cardiovagal BRS may support the maintenance of stable hyperthermic circulation with high cardiac output and elevated arterial pressure, despite reduced peripheral vascular resistance. In contrast, Li, *et al*.^3^ postulated that low BRS is responsible for circulatory collapse during passive hyperthermia. Confirming this hypothesis, improvement of baroreflex with thermal preconditioning, which induced Hsp70 expression in the nucleus tractus solitarii, attenuated bradycardia and hypotension during heatstroke in anaesthetized rats^3^.

In contrast, an increase in BRS was reported in a study where the sensitivity of the bradycardic response to an elevation in arterial pressure was augmented in baboons at 39.5 °C, although BRS derived from the whole pressure-heart rate curve was not changed^6^. A recent study in young healthy men^7^ provided evidence that resetting of the baroreflex to adjust to an elevated heart rate during hyperthermia was associated with increased cardiovagal BRS and amplified the capacity to respond to elevated arterial pressure with bradycardia. Simultaneously, the baroreflex control of arterial pressure was well preserved. These observations may indicate that efficient circulatory control through the baroreceptor reflex is required for successful adaptation to passive hyperthermia, and conditions characterized by decreased baroreflex sensitivity (old age, heart failure, hypertension, obesity, and diabetes mellitus) may be associated with reduced thermotolerance. This hypothesis was experimentally demonstrated in rats with sinoaortic denervation^2^. In these rats, the sympathomimetic effect of passive hyperthermia was amplified, the speed of the Tc increase was accelerated, and thermotolerance was reduced. Thus, increased BRS, in response to rising Tc, could represent an adaptive mechanism to limit sympathetic activation and reduce the speed of elevation of Tc, as documented by Stauss and colleagues^8^.

To address these controversies, improve the understanding of the pathogenesis of arterial pressure decline during passive hyperthermia, and provide a better framework for potential interventions, we tested the hypothesis that changes in cardiovagal BRS (sensitization as well as desensitization) during passive hyperthermia depend on Tc, and that the absence of the cardiovagal baroreflex is closely related to the onset of arterial pressure decline in terms of time. We used rats with implanted telemetric transmitters to non-invasively monitor ECG, aortic blood pressure, core body temperature and locomotor activity. Cardiovagal BRS was derived from spontaneous fluctuation of R-R intervals and systolic aortic pressure with 1 s resolution using the procedure introduced by Eckberg and Kuusela^9^.

## Results

### Haemodynamic response to passive hyperthermia

**Figure 1:**
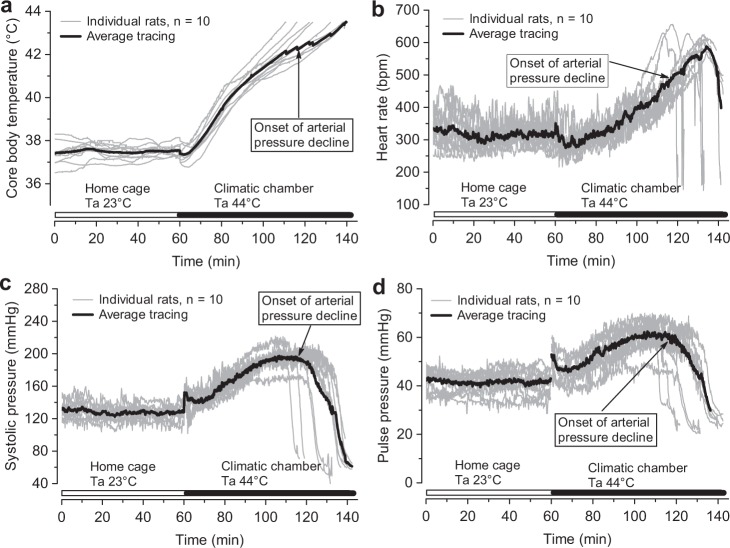
Core body temperature and haemodynamic changes during passive hyperthermia. Tracings during the first 60 min were recorded in the animals’ home cages at an air temperature (Ta) of 23 °C. Then, the rats were transferred to a climatic chamber with a Ta of 44 °C. Original data were averaged over 10-s intervals for plotting. Elevated Ta values induced gradual elevation in core body temperature, i.e., passive hyperthermia (**a**), and biphasic responses of heart rate (**b**), systolic (**c**), and pulse (**d**) pressures.

Placing animals into the climatic chamber with a Ta of 44 °C initiated the gradual elevation of Tc accompanied by an increase in systolic pressure and heart rate (Fig. 1). However, these changes occurred with a 10- to 20-min delay, during which the monitored parameters were rather stable, with the exception of an initial short stress response associated with the handling of animals. Arterial pressure started to decline at a Tc of 42.5 (0.3) °C (Fig. 1c). When the arterial pressure began to decline, the heart rate continued to increase and started to decrease rapidly at a Tc of 43.3 (0.3) °C (Fig. 1b). The delay between the onsets of decline in the arterial pressure and the heart rate was 11.9 (3.1) min (p = 10^−6^, n = 10, paired t-test).

### Temperature-dependent changes in BRS

**Figure 2:**
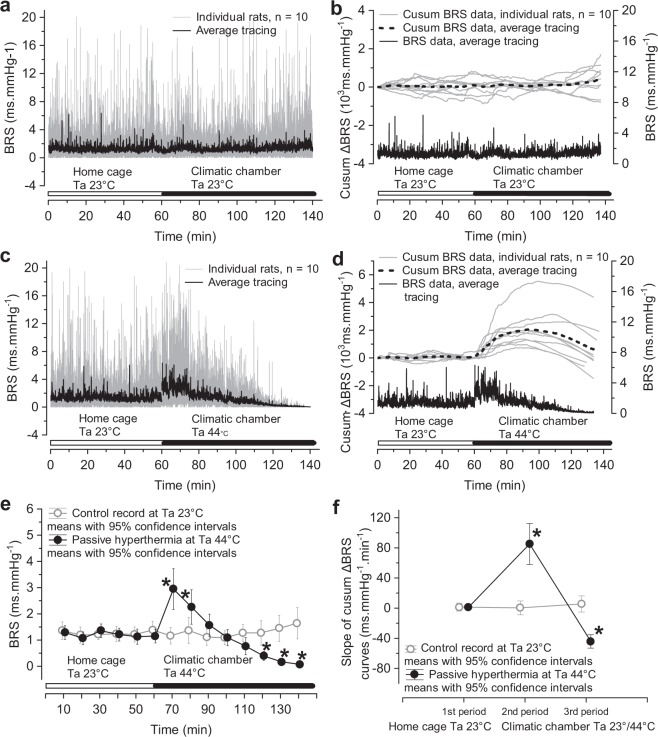
Time-course of changes in cardiovagal baroreflex sensitivity (BRS) during passive hyperthermia. BRS was estimated in 10-s windows and moved in 1-s steps as a transfer function modulus between systolic pressure and R-R interval fluctuations in the low frequency band of R-R interval oscillations (0.2–0.7 Hz) with a coherence limit of 0.5 and negative phase shift. BRS in the home cage and in the climatic chamber at an air temperature (Ta) of 23 °C (**a**). Cumulative sum of deviations of the actual BRS from the mean BRS (cusum ΔBRS) in the home cage at a Ta of 23 °C (**b**). BRS in the home cage at a Ta of 23 °C and in the climatic chamber at a Ta of 44 °C (**c**). Cusum ΔBRS at Ta values of 23 °C and 44 °C (**d**). Mean BRS values of 10-min bins (**e**) from data displayed in (**a**,**c**). A univariate test for repeated measures ANOVA with Greenhouse-Geisser correction was used to analyse the effects of time spent in the climatic chamber and of Ta on BRS. Interaction time vs. Ta: F(3.79, 34.14) = 21.303, p < 0.0000001, ε = 0.292, partial η^2^ = 0.703. Pairwise comparisons of BRS at Ta 23 °C vs. 44 °C with the Bonferroni test: *p < 0.0166. Panel (**f**) displays slopes of cusum ΔBRS curves from panels (**b**,**d**). The 1^st^ period relates to the record in the home cage at a Ta of 23 °C, 2^nd^ period to the ascending limb and 3^rd^ period to the descending limb of the cusum ΔBRS curves recorded in the climatic chamber at a Ta of 44 °C. Wilks multivariate test for repeated measures ANOVA was used to determine the effects of time (periods of passive heating) and Ta on changes in the slopes of cusum ΔBRS curves: interaction time (periods) vs. Ta: F(2,8) = 76.226, p = 0.000006, n = 10; partial η^2^ = 0.886; pairwise comparisons of slopes at Ta values of 23 °C and 44 °C with the Bonferroni test: *p < 0.00014.

Our data showed that BRS in rats was not constant but permanently oscillated (Fig. 2a,c). BRS was highly variable within the range of near zero values up to 20 ms.mmHg^−1^. The distribution of BRS values was skewed to the right, with a larger proportion of low BRS values. Because one high BRS value temporarily coincided with several rather low BRS values, averaging of the data of all 10 rats removed the high BRS values; the average BRS was mostly below 2 ms.mmHg^−1^, which is a common finding in rats. The oscillations of BRS values were concentrated (65% of the total power) in the frequency band of 0.02–0.2 Hz, which may correspond to BRS fluctuations in the very-low-frequency band described in humans^9^. Thus, estimation of an average BRS during a longer period may not reflect possible alterations in BRS during the inherently non-stationary condition of hyperthermia. Therefore, we identified possible trend changes in BRS during passive hyperthermia with the cusum procedure (Fig. 2b,d). Permanently increased or decreased deviations from the reference value (mean BRS in home cages) produce lines with positive or negative slopes on the cusum plots^10^.

**Figure 3:**
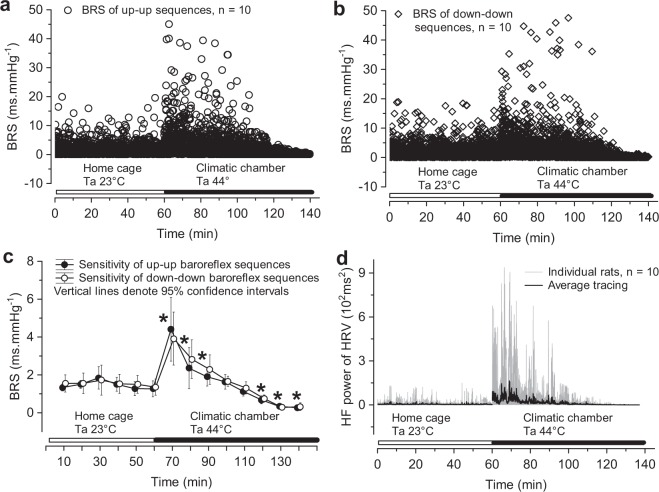
Effect of passive hyperthermia on cardiovagal baroreflex sensitivity (BRS) estimated with the sequence method. BRS of sequences of increased systolic pressure and prolonged R-R intervals (**a**). BRS of sequences of declining systolic pressure and shorter R-R intervals (**b**). Mean BRS values of 10-min bins (**c**) from data displayed in (**a**,**b**). Effect of time and ambient temperature (Ta) on BRS (up-up and down-down sequences together) was analysed with adjusted (Greenhouse-Geisser) univariate tests for repeated measures ANOVA: F(2.603, 28.624) = 34.558, p < 0.000001, ε = 0.2002, partial η^2^ = 0.759. Pairwise comparisons of BRS in the climatic chamber with the average BRS in the home cage were performed with the univariate test of significance for planned comparisons and the Bonferroni correction, *p < 0.00625. High-frequency (HF) power of heart rate variability (HRV), a surrogate measure of parasympathetic drive to the sinoatrial node (**d**).

Transferring animals into the climatic chamber at 23 °C did not change the BRS (Fig. 2a,b,f). However, when the Ta in the climatic chamber was 44 °C, BRS initially increased and later declined (Fig. 2c,d,f). The BRS averaged over arbitrarily chosen 10-min bins confirmed the initial baroreflex sensitization followed by desensitization (Fig. 2e). The BRS data in Fig. 2 represent the transfer function modulus between the systolic pressure and R-R interval time series^11^. To substantiate the results, we also used the sequence method of BRS estimation^12^, which has been validated for use in rats^13^. Figure 3(a–c) shows that the BRS derived with the sequence technique behaved similarly to the transfer function modulus BRS. An abrupt elevation of high-frequency power of heart rate variability upon exposing animals to a Ta of 44 °C suggests that baroreflex sensitization could be explained by augmented parasympathetic activity (Fig. 3d).

**Figure 4:**
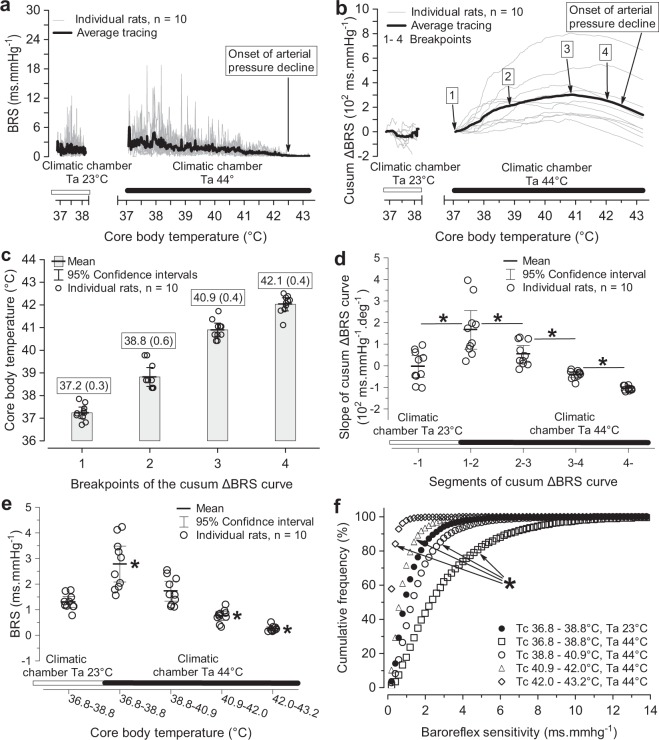
Dependence of cardiovagal baroreflex sensitivity (BRS) on core body (Tc) and air (Ta) temperature. BRS before and during passive hyperthermia plotted against Tc (**a**). Cumulative sum of BRS deviations from the mean BRS in the climatic chamber at a Ta of 23 °C (cusum ΔBRS) plotted against Tc (**b**). Breakpoints in the trend of BRS values were identified with MARS. Breakpoint 1 coincides with placing animals into the climatic chamber heated to a Ta of 44 °C. The segment (1–2) corresponds to the period when the Tc of the animals was within the normal range, but BRS was higher than that at a Ta of 23 °C; during the segment (2–3), Tc is increasing and BRS is lower than that in the segment (1–2) but still above BRS at a Ta of 23 °C; after breakpoint 3, BRS is below the reference value; after breakpoint 4, BRS is near zero. Tc at structural breakpoints of the cusum ΔBRS curves (**c**). The numbers in brackets are standard deviations. Panel (**d**) shows slopes of segments of cusum ΔBRS curves from the panel (**b**). Significance of changes in the slopes of segments of cusum ΔBRS curves: F(4,6) = 16.0743, p = 0.002328, n = 10, partial η^2^ = 0.7272 (Wilks multivariate test for repeated measures). Pairwise comparison of slopes: *p < 0.005 (univariate test for planned comparisons). Changes in slope values followed a quadratic trend: F(1,9) = 24.9518, p < 0.000744, n = 10. Panel (**e**) displays BRS values averaged over segments of cusum ΔBRS curves identified in the panel (**b**). The significance of BRS changes during exposure to a Ta of 44 °C: F(4,6) = 49.246, p = 0.000101, n = 10, partial η^2^ = 0.7316 (Wilks multivariate test for repeated measures). Pairwise comparison of BRS at a Ta of 44 °C vs. BRS at a Ta of 23 °C: *p < 0.0065 (univariate test for planned comparisons). Changes in BRS values followed a quadratic trend: F(1,9) = 31.4621, p = 0.00033. Panel (**f**) shows distribution of BRS values in the segments of cusum ΔBRS curves identified in the panel (**b**). Significance of the shift in curve position: *p < 0.0001 (two-sample Kolmogorov-Smirnov test).

To identify how changes in BRS depend on Tc, we resampled the BRS data and constructed BRS (Fig. 4a) and cusum ΔBRS (Fig. 4b) versus Tc curves. The first break point in the trend of BRS coincided with the exposure of animals to hot air (Ta of 44 °C) and heralded an abrupt increase in BRS, which was associated with no or minimal elevation in Tc. The next break point was identified at a Tc of 38.8 (0.6) °C. After this Tc, BRS started to decline but remained above the reference values estimated in the home cage at a Ta of 23 °C. After a Tc of 40.9 (0.4) °C, BRS progressively declined below the reference value. The reduction in BRS was further amplified after reaching a Tc of 42.1(0.4) °C, and the baroreflex was virtually non-operative (Fig. 4b–e).

The effects of Ta and Tc on the distribution of BRS values is shown in Fig. 4f. In response to hot air (a Ta of 44 °C), the distribution of BRS shifted towards higher values. As the Tc increased, the proportion of high BRS values returned to the baseline distribution. Further Tc elevation was associated with expanded proportion of low BRS values.

### Interdependence of BRS, haemodynamic parameters, and Tc

**Figure 5:**
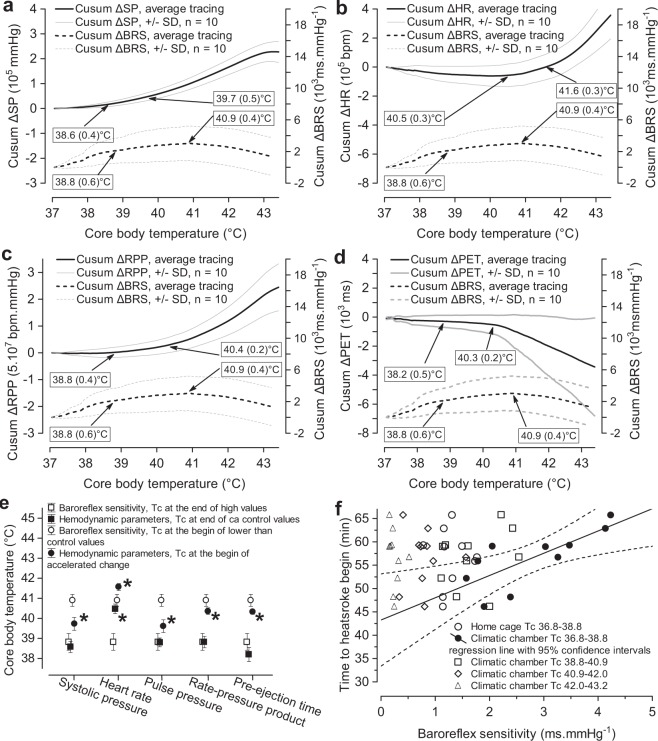
Trend changes in haemodynamic parameters during passive hyperthermia. Systolic pressure (SP, **a**), heart rate (HR, **b**), rate-pressure product (RPP, **c**), pre-ejection time (PET, **d**) and cardiovagal baroreflex sensitivity (BRS) are plotted against the core body temperature (Tc), as a cumulative sum of deviations (cusum Δ) from the means estimated during the stay in the home cage at an air temperature (Ta) of 23 °C. Breakpoints on cusum curves (arrows, numbers in quadrilaterals) were identified with multivariate adaptive regression splines (n = 10). The period with elevated BRS values ended at a Tc of 38.8 (0.6) °C, and the period with baseline BRS values ended at a Tc of 40.9 (0.4) °C. Breakpoints on haemodynamic cusum curves denote the end of the initial period with approximate baseline values and the beginning of the period characterized by accelerated changes in haemodynamic parameters. The numbers in brackets are standard deviations. Tc values at which trend changes in BRS and haemodynamic parameters occurred were compared with multivariate tests for repeated measures (Wilks) separately for each haemodynamic parameter (**e**). Univariate tests of significance for planned comparison with the Bonferroni correction were used for pairwise comparisons of corresponding breakpoints on BRS and haemodynamic cusum curves. Whiskers denote 95% confidence intervals, *p < 0.01539 (**e**). Linear regression line between the time to the onset of arterial pressure decline and increased BRS during the initial contact with hot air: y = 43.257 + 4.769 × , r^2^ 0.5713, p = 0.01144. Linear relationships between the time to the onset of arterial pressure decline and BRS during the stay in the home cage or BRS at elevated Tc in the climatic chamber were not significant: r^2^ = 0.0016, p = 0.913, (**f**).

The decline of BRS during passive hyperthermia may affect haemodynamic stability. Figure 5 shows that during the first period of passive hyperthermia, characterized by a not-yet-elevated Tc and augmented BRS, the systolic pressure remained stable and the heart rate actually declined below the baseline reference value. Surrogate measures of oxygen consumption (i.e., rate-pressure product) and sympathetic drive to the heart (i.e., pre-ejection time) also exhibited cusum curves with minimal slopes. During the subsequent period (rising Tc, declining BRS), the change in haemodynamic parameters accelerated, with the exception of heart rate, which remained below the reference value. The next trend change in haemodynamic parameters occurred at a Tc of 40–41 °C. However, the Tc breakpoints for haemodynamic parameters were significantly lower than the Tc-breakpoint for the acceleration of BRS decline (Fig. 5e). Heart rate was an exception, showing augmentation only after a Tc of 41.6 (0.3) °C (Fig. 5e). A regression analysis showed that the maximal BRS reached during the initial period of exposure to hot air predicted the onset of arterial pressure decline (Fig. 5f).

### Pharmacologically-induced cardiovagal baroreflex desensitization

**Figure 6:**
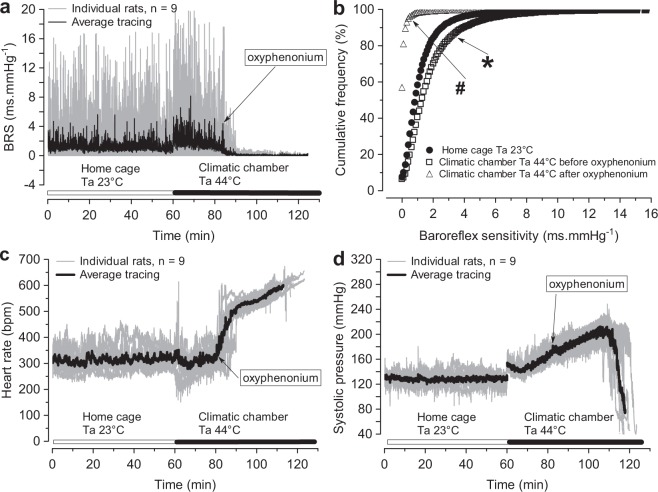
Pharmacological cardiovagal baroreflex desensitization at a core body temperature (Tc) of 40 °C. Muscarinic blockade with oxyphenonium bromide (10 µmol.kg^−1^) via i.p. injection at a Tc of 40 °C caused abrupt reduction of cardiovagal baroreflex sensitivity (BRS) to values near zero (**a**). A corresponding shift in the distribution of BRS values (**b**). The cumulative frequency of BRS values estimated in the home cages at an ambient air temperature (Ta) of 23 °C was different from the BRS cumulative frequency curve in the climatic chamber at a Ta of 44 °C before muscarinic blockade (*p < 0.001, two-sample Kolmogorov-Smirnov test). Oxyphenonium administration caused a significant (^#^p < 0.001, two-sample Kolmogorov-Smirnov test) change in the distribution of BRS towards low values (**b**). Muscarinic blockade instantly elevated the heart rate (**c**) but had no effect on systolic pressure (**d**).

To confirm or reject the postulated negative effect of reduced cardiovagal BRS on haemodynamic stability during passive hyperthermia, we applied the parasympatholytic drug oxyphenonium at a Tc of 40 °C to abruptly abolish baroreflex regulation of the heart rate during the period of augmented BRS (Fig. 6a,b). Oxyphenonium elevated the heart rate from 329 (21) bpm to 516 (31) bpm within 5 min after the i.p. injection (p < 0.001, n = 9, paired t-test) (Fig. 6c). However, virtually no effect of parasympatholysis and baroreflex desensitization on systolic pressure was seen (Fig. 6d).

## Discussion

Haemodynamic changes induced with passive hyperthermia in our unrestrained rats with telemetric implants showed a similar pattern as previously presented data^14,15^, i.e., an increase in the Tc associated with the elevation of arterial pressure and progressive tachycardia. However, the alterations in the haemodynamic parameters were not linear. In particular, during the initial period, the arterial pressure was relatively stable and the heart rate temporarily even declined, which could reflect well-functioning baroreflex regulation. In the course of ongoing exposure to a hot environment, the arterial pressure progressively increased, following an S-shaped saturation curve, until it began to decline. Concurrently, the heart rate exhibited rather exponential growth kinetics, which was possibly caused by gradually decreased baroreflex regulation together with the augmented impact of increasing Tc and adrenal medullary secretion. In representative plots by Quinn, *et al*.^16^, similar kinetics of systolic pressure and heart rate changes during passive hyperthermia were observed.

At the onset of arterial pressure decline, tachycardia continued to rise. This finding excludes the heart rate decrease as a cause of the onset of arterial pressure decline and is rather suggestive of an active baroreflex mechanism. However, because cardiovagal BRS was low when arterial pressure began to decline, augmented tachycardia most likely resulted from the direct effect of elevated Tc^17^ and baroreflex-independent activation of the sympathetic-adrenal medullary and hypothalamic-pituitary-adrenocortical systems^18,19^. Our data could not exclude some contribution of a potentially functional cardiosympathetic baroreflex. Heart rate reached its maximum above a Tc of 43 °C, suggesting that the cardiac excitatory-conductive system is highly resistant to thermal damage. Sudden bradycardia above 43.5 °C is best explained by the injury of pacemaker cells of the sinoatrial node and hyperthermic general organ failure, which includes myocardial ischaemia^20^.

Data from anaesthetized rats are contradictory. While Kielblock and colleagues^21^ documented a similar haemodynamic response to heat stress as described in conscious rats, in experiments conducted by Li *et al*.^3^, the onset of arterial pressure decline was associated with progressive bradycardia. This finding led to the hypothesis that bradycardia caused by a failing baroreflex function is responsible for this arterial pressure decline. Consistent with this hypothesis, potentiation of baroreceptor reflex capacity and sensitivity prevented hyperthermic haemodynamic collapse^3^. However, in conscious rats, which exhibit tachycardia at the onset of arterial pressure decline, the mechanism of decrease in arterial pressure must be different and is likely mediated by reduced splanchnic vascular resistance and stroke volume^21,22^. The reduction in pulse pressure at the onset of arterial pressure decline (Fig. 1d) suggests a decrease in the stroke volume and the speed of ejection, i.e., myocardial failure. Augmentation of the pulse pressure by the postulated decrease in the splanchnic and total vascular resistance is likely counterbalanced by tachycardia and an increase in the vascular compliance.

Haemodynamic adaptation to a hot environment is intrinsically a non-stationary process. Thus, we selected a cross-spectral estimation of BRS using a sliding-short window fast Fourier analysis of systolic pressure and heart rate variability^9^, which allows BRS assessment on a nearly continuous timescale. This time-resolved analysis showed that spontaneous cardiovagal BRS in rats permanently fluctuates, similar to humans^9,23^, although with a higher frequency of 0.02–0.2 Hz. Cusum charts (Fig. 2b,d) documented that baseline BRS fluctuations were significantly altered in the hot air environment. Initial elevation of BRS (rising segment of the cusum curve) was replaced with a period of BRS gradually declining towards baseline values (low slope to horizontal part of the cusum curve) and eventually with BRS values below the baseline (declining arm of the cusum curve). All rats responded in the same way, although large differences existed with respect to the sizes of BRS changes. We used the MARS analysis to identify Tc values at which the trend in BRS changed (Fig. 4b). Initially, elevated BRS was measured when Tc was within the range of 36.8–38.8 °C, i.e., normal or slightly elevated. Thus, the baroreflex sensitization at the beginning of thermal stress seems not to be related to changes in Tc but to the high Ta of 44 °C. The structural breakpoint in the BRS-cusum curve at a Tc of 38.8 (0.6) °C ends the period of high BRS (steep rising slope of the cusum curve). Beyond this breakpoint, BRS receded from its maximal values (lower rising slope on the cusum curve) but remained above the reference baseline BRS. The next breakpoint at a Tc of 40.9 (0.4) °C marked the phase with below-baseline BRS, which may coincide with the appearance of the first signs of thermal damage to other organ systems, such as the liver, intestine^20^ or brain^24^. Thermal cardiovagal baroreflex desensitization was accelerated with increasing Tc, and above a Tc of 42.1 (0.4) °C, the cardiovagal baroreflex was virtually non-operative in heart rate control.

Our experiment confirms the finding that cardiovagal BRS is reduced at a Tc of 41.5 °C^4^. Because the breakpoint between elevated and reduced BRS was observed at a Tc of 40.9 (0.4) °C, our results do not necessarily contradict the data published by Stauss and colleagues^8^, which showed elevated BRS at a Tc of 40–41 °C. Although the kinetics of BRS changes during passive hyperthermia documented by Stauss, *et al*.^8^ differ from our average BRS trend, three of our rats exhibited BRS changes as described by Stauss, *et al*.^8^, i.e., Tc-dependent gradual accentuation of BRS, with the maximum effect observed at 40–41 °C (Fig. 4b).

While we have shown that the baroreflex controls the heart rate up to a Tc of 41.6 (0.3) °C (Fig. 5b), our data do not support the importance of heart rate control and functions mediated by muscarinic receptors for haemodynamic stability during passive hyperthermia. Concerns related to a strongly elevated heart rate are three-fold. High heart rates may reduce stroke volume by limiting the ventricular filling time and jeopardizing the myocardium due to an augmented oxygen demand and simultaneously reduced coronary perfusion. However, instantaneous peripheral muscarinic blockade with oxyphenonium at a Tc of 40.0 °C did not affect arterial pressure stability despite abrupt abolition of the cardiovagal baroreflex and high tachycardia (Fig. 6). These results support the contention that low BRS may help achieve the maximal heart rate required to maintain stable circulation at high Tc values, when a large cardiac output and high arterial pressure are present^4^. Nevertheless, some shortenings of the time to the onset of arterial pressure decline were detected after oxyphenonium administration (56.5 (6.2) min, n = 10 vs. 51.8 (3.1) min, n = 9, p = 0.0519, t-test), but this finding is best explained by the inhibition of salivation^25^.

During the initial period with strongly elevated BRS, arterial pressure and surrogate measures of oxygen consumption (the rate-pressure product) and sympathetic drive to the heart (pre-ejection time) remained near baseline levels. However, the Tc at which trend changes in arterial pressure, the rate-pressure product or pre-ejection time occurred was lower than the Tc breakpoints in BRS development (Fig. 5e), which also does not support the direct cause-effect relationship between cardiovagal baroreflex desensitization and haemodynamic alterations during passive hyperthermia. Similar Tc values for acceleration of arterial pressure increases (39.7 (0.5) °C) and pre-ejection time shortening (40.3 (0.2) °C) implicate sympathetic-adrenal medullary activation as a possible cause of hyperthermic arterial hypertension.

Although our data do not support the direct involvement of cardiovagal desensitization in the induction of arterial pressure decline, the fact that cardiovagal BRS during the initial period of passive hyperthermia at a Ta of 44 °C explained 57% (p = 0.01144) of the variability in the time to the onset of arterial pressure decline (Fig. 5f) deserves attention. Since spontaneous BRS mainly reflects parasympathetic control of the heart rate^13,26^, the initial elevation in BRS may be interpreted as a sign of augmented parasympathetic drive to the heart possibly initiated by the noxious, irritant effect of hot air on the trigeminal sensory nerves in the face and airways^27,28^. This conclusion is underpinned by the augmented power in the high-frequency band of heart rate variability immediately after exposing the animals to a hot environment (Fig. 3d). Coincident with parasympathetic activity, the sympathetic drive to the heart was also elevated (Fig. 5d), supporting activation of the trigeminal protective reflexes (nasopharyngeal or startle reflexes) as a possible mechanism of initial autonomic co-activation^27^ in response to hot air.

While simultaneous activation of both branches of the autonomic nervous system is not a rare response to a stressor^27^, this effect has not yet been described during heat stress; instead, parasympathetic withdrawal has been consistently documented^29–35^. We have shown that transitional parasympathomimetic effect was not present when the ambient air temperature was not elevated, and it was not related to the elevation in Tc. Thus, the proposed autonomic nervous system co-activation in response to airway stimulation by hot air could remain unseen if the monitoring did not include the initial period of heat exposure^35^, Ta was below the value needed for activation of heat sensitive channels (TRPV1)^16^, i.e. below 42–43 °C^36^, or airway sensory receptors were not exposed to the elevated ambient temperature, i.e. during whole-body heating, which excludes the face and airways from heat exposure^1^. Exposure to high Ta values greater than 70 °C, such as in a sauna, may override stimulation of the parasympathetic system by activation of nociceptive reflexes^37^.

Limitations. We used transfer function method to estimate spontaneous BRS during 10-s sliding windows. This method involves several assumptions^38^, but we believe that the results reflect true changes in BRS during passive hyperthermia because similar alterations were identified with a mathematically different approach of the sequence method (Fig. 3). Our experiment was designed to describe the haemodynamic response to passive, non-interrupted hyperthermia in conscious, undisturbed animals. Therefore, additional interventions that may confirm an increase in parasympathetic drive to the sino-atrial node and possibly to other vagally innervated structures, were not possible. A positive association between the initial baroreflex sensitization and the time to the onset of arterial pressure decline was rather modest (r = 0.7559, 95% CI: 0.2409–0.9387, p = 0.0114). In addition, data in the lower BRS range showed a larger spread of residuals (Fig. 5f) and should be interpreted more cautiously. Because of the limitations related to the design of the experiment, we could not provide any evidence for the possible causality/mechanism (if one exists) behind the observed positive association between BRS and the onset of arterial pressure decline. However, this association is in line with the finding that accelerated sympathetic activation, heart rate and arterial pressure elevation in rats with sinoaortic deafferentation during passive hyperthermia are associated with reduced thermal tolerance^2^. We can only speculate that simultaneous activation of both branches of the cardiac autonomous system may help efficiently adjust the cardiac pumping function^39^. In addition, a generalized increase in parasympathetic activity, if present, may mitigate^40^ the onset of systemic inflammatory response syndrome^20^ during heatstroke as well as suppress intestinal inflammation^41^ and increase in gut permeability^42^.

In conclusion, increasing core body temperature desensitizes the cardiovagal baroreflex. This desensitization does not seem to be related to the onset of arterial pressure decline. However, depending on the heating conditions, cardiovagal baroreflex sensitivity can be temporarily elevated, probably due to increased parasympathetic activity. Initial cardiovagal baroreflex sensitization was positively associated with the time to the onset of arterial pressure decline, which may indicate a possible protective role of early parasympathetic activation during exposure to a hot environment.

## Methods

### Ethical approval

The Animal Ethics Committee at the Health Science Center of Kuwait University approved the experiments. Handling of experimental animals complied with the National Institutes of Health’s Guide for the Care and Use of Laboratory Animals (8^th^ edition). The Animal Resources House of the Health Science Center of Kuwait University, which was responsible for supervising adherence to the approved procedures, supplied the animals.

### Experimental animals and housing

Twelve-week-old male Wistar-Kyoto rats were individually housed in polycarbonate cages (22 × 38 × 20 cm) with corncob bedding (Shepherd’s Cob, Shepherd Specialty Papers, Milford, NJ, USA). The animal room temperature was maintained at 23 (0.7) °C with a 12:12-h light/dark cycle (lights on at 6 p.m., off at 6 a.m.). The 5LF2 EURodent Diet 14% (LabDietPMI Nutrition International, Brentwood, MO, USA) and tap water were provided ad libitum.

### Implantation of telemetric transmitters

HD-S11 (Data Sciences International, St. Paul, MN, USA) transmitters were implanted under general anaesthesia with intraperitoneal (i.p.) injection of 120 mg.kg^−1^ ketamine hydrochloride (Tekam 50, Hikma Pharmaceuticals, Amman, Jordan), 6 mg.kg^−1^ xylazine hydrochloride (Sigma-Aldrich Chemie, Taufkirchern, Germany) and 0.12 mg.kg^−1^ atropine sulphate monohydrate (Sigma-Aldrich Chemie, Taufkirchern, Germany). The depth of anaesthesia was tested with corneal reflex and foot pressure sensitivity. If needed, an additional dose of ketamine/xylazine was given. Eyes were protected with an ophthalmological ointment, and body temperature was controlled at 36 °C with a rectal probe and heating pad. Diclofenac sodium (10 mg.kg^−1^, i.p.) (Sigma-Aldrich Chemie, Taufkirchern, Germany) was applied as a postsurgical analgesic for two days. The body of the telemetric implant was placed intra-abdominally. The tip of the pressure catheter was positioned in the abdominal aorta above the bifurcation through the femoral artery. The negative ECG electrode was placed into the upper mediastinum, and the positive electrode was fixed on the surface of the thorax below the thoracic muscles in the left midclavicular line^43^.

Successfully implanted animals (n = 20) were randomly assigned (with QuickCalcs software, GraphPad Software, La Jolla, CA, USA) to 2 experimental groups, each with n = 10 animals. Data recorded in the control group served to describe BRS changes during passive hyperthermia. To abruptly and fully desensitize the baroreflex, the animals in the second group received oxyphenonium bromide (Sigma-Aldrich, Chemie, Taufkirchern, Germany; 10 µmol.kg^−1^ in phosphate-buffered saline) at a Tc of 40 °C. Heating was interrupted for less than 20 s to administer oxyphenonium via i.p. injection. One implant failed in the oxyphenonium group; thus, data from only 9 animals were available.

### Heating protocol

Experiments started 4 weeks after surgery, when the animals had fully recovered, as verified by eating and drinking patterns, behaviour, weight gain and cardiovascular circadian rhythms. Passive hyperthermia was induced in the climatic chamber with an air temperature (Ta) of 44 (0.1) °C and humidity of 13 (2)%. This Ta was selected because it is a common air temperature in many inhabited areas and was consistently used in comparable experiments^4,8^. Rats subjected to the hot environment felt discomfort but not pain^24^. During the procedure, the rats were placed in polycarbonate cages (23 × 18 × 15 cm) with perforated walls and no access to food or water. To minimize the unspecific startle response, rats were adapted to the experimental conditions by placing them into the climatic chamber at a Ta of 23 °C for 1 h three times. After the adaptation, baseline recording, which included 2 h of monitoring in home cages in the animal room followed by 2 h in the climatic chamber at a Ta of 23 °C, was performed. The next day, the procedure was repeated, but the Ta in the climatic chamber was 44 °C. When the systolic pressure decreased to 50 mmHg, the animals were euthanized with an overdose of thiopentone sodium (200 mg.kg^−1^, i.p., Intraval Sodium, May & Baker, Dagenham, England). To standardize the content of the gut, food was removed 16 h before, and water 1 h before, placing the animals into the climatic chamber.

### Data collection and analysis

Aortic pressure, ECG, Tc and locomotor activity were sampled at 1 kHz with Dataquest A.R.T. version 4.36 software (Data Sciences International, St. Paul, MN, USA). Original waveforms were imported into WinCPRS version 1.162 software (Absolute Aliens, Turku, Finland), which was used to derive R-R intervals; systolic, diastolic, and pulse pressures; pre-ejection time; rate-pressure product; and spontaneous cardiovagal BRS. To estimate BRS with a time resolution of 1 s, we used the procedure described by Eckberg and Kuusela^9^, Eckberg *et al*.^44^ adjusted to rats. The transfer function between systolic pressure and R-R interval changes was calculated in the low-frequency band (0.2–0.7 Hz) with a coherence limit > 0.5, and phase angle < 0 in the 10 s windows, moved by 1-s steps through the whole data collection period. Non-segmented fast Fourier transform with a Hanning window was used to derive systolic pressure and R-R interval spectra. Systolic pressure and R-R interval time series were resampled at 30 Hz and the 1^st^ order polynomial baseline was removed. The 10-s window that includes 2–7 full oscillation in the 0.2–0.7 Hz frequency band was chosen as a compromise between the frequency resolution and the time resolution needed to estimate expected BRS changes in the highly non-stationary condition of passive hyperthermia. Parasympathetic drive to the heart was assessed as R-R interval spectral power in the high-frequency band of 0.7–3 Hz. BRS was also assessed with the sequence method^45^. Whole systolic pressure and R-R interval time series were searched for up-up and down-down sequences of at least 3 consecutive beats with a lag of 2 beats. Only sequences with a minimum systolic pressure change of 0.1 mmHg, a minimum R-R interval change of 0.1 ms, and a minimum correlation of 0.85 were accepted. The onset of arterial pressure decline was defined as the moment with the highest systolic pressure in the time series of 5-s systolic pressure averages. The hypotensive period was the interval between the onset of arterial pressure decline and the time when systolic pressure decreased to 50 mm Hg.

### Statistical analysis

Data are presented as the arithmetic means with standard deviations in brackets. Several different statistical procedures were used: a univariate test for repeated measures ANOVA with Greenhouse-Geisser adjustment, the Wilks multivariate test for repeated measures ANOVA, one-way ANOVA with trend analysis, the Bonferroni post hoc test for pairwise comparisons, a univariate test of significance for planned comparisons with Bonferroni correction, a two-sample Kolmogorov-Smirnov test, and regression analysis. Details about the hypotheses tested and statistical procedures used, and their results are presented in the figure legends. When required, the probability (p > 0.05) of a non-normal distribution of the analysed data was assessed with Shapiro-Wilk’s W test. When multiple comparisons were performed, Bonferroni correction was used to adjust the p-values. Statistical analysis was performed with Statistica version 12 (Stat Soft, OK, USA). Breakpoints on cumulative sum (cusum) curves were identified with multiple adaptive regression splines (MARS) using SPM version 8.2 software (Salford Systems, CA, USA).

## Data Availability

The original recorded waveforms (in the binary Dataquest A.R.T format) and datasets (in WINCPRS and Statistica formats) generated during the current study are available from the corresponding author on reasonable request.
